# Pruritus Associated with Commonly Prescribed Medications in a Tertiary Care Center

**DOI:** 10.3390/medicines6030084

**Published:** 2019-08-04

**Authors:** Amy H. Huang, Benjamin H. Kaffenberger, Adam Reich, Jacek C. Szepietowski, Sonja Ständer, Shawn G. Kwatra

**Affiliations:** 1 Department of Dermatology, Johns Hopkins University School of Medicine, Baltimore, 21205 MD, USA; 2 Bloomberg School of Public Health, Johns Hopkins University, Baltimore, 21205 MD, USA; 3 Division of Dermatology, Ohio State University Wexner Medical Center, Columbus, 43210 OH, USA; 4 Department of Dermatology, University of Rzeszow, 35-310 Rzeszow, Poland; 5 Department of Dermatology, Venereology and Allergology, University of Medicine, 50-367 Wroclaw, Poland; 6 Department of Dermatology, University Hospital of Münster, 48149 Münster, Germany

**Keywords:** pruritus, itch, drug-induced, medication-related, epidemiology

## Abstract

**Background:** Sparse data are available on rates of drug-induced pruritus, a well-recognized adverse reaction. We sought to assess relative rates of pruritus associated with commonly prescribed medications. **Methods:** Using the electronic medical record system EPIC, retrospective data were collected on patients seen at Johns Hopkins who received a medication of interest in a five-year period (2013–2018). Sequential criteria were used to identify the subpopulation who presented with a chief complaint of “pruritus” or diagnosis of “itching” within three months of receiving drugs. **Results:** We identified 9802 patients with pruritus after drug initiation and 1,085,404 patients without. A higher proportion of those with pruritus were female (70%) than those without (58%), *p* < 0.001. Patients in both groups were most commonly 50 to 79 years old. A higher proportion of patients with pruritus were black (40%) compared to those without (23%), *p* < 0.001. In this study, the highest rates of pruritus were observed with heparin (1.11%), trimethoprim-sulfamethoxazole (1.06%), and calcium channel blockers (0.92%). Psychiatric/neurologic drugs used to treat pruritus were associated with low rates of itch. **Conclusions:** Certain cardiovascular and antimicrobial agents are associated with increased frequencies of pruritus. This knowledge may guide providers in clinical selection of commonly used agents to minimize adverse effects associated with reduced compliance.

## 1. Introduction

Drug-induced pruritus is a well-recognized adverse reaction, accounting for more than 10% of cutaneous drug reactions in previous studies [1]. Depending on the causative agent, symptoms may be acute in onset and resolve upon drug cessation or evolve into chronic pruritus lasting more than six weeks [1]. Skin lesions may or may not be visible as pruritus can result directly from inflammation of the skin or indirectly through systemic mediators, such as cholestatic liver injury [2,3]. In addition to negatively impacting quality of life, drug-induced pruritus has been associated with decreased medication compliance [4]. Despite this, sparse data are available on the association of pruritus with many commonly used medications. Previous studies have been limited to small case series or narrow in scope by focusing on a single healthcare setting or a single drug/drug class. In the present study, we use longitudinal data from an integrated health system to assess relative rates of pruritus associated with a variety of widely prescribed medications.

## 2. Materials and Methods

Institutional review board approval was waived because only anonymous aggregate-level data were used. Retrospective data on inpatient, outpatient, and emergency department (ED) visits were collected using the SlicerDicer feature of EPIC, the electronic medical record system of Johns Hopkins Health Systems (JHHS) [5]. The JHHS network includes tertiary care and community-based hospitals as well as a network of outpatient primary care/specialty clinics. In addition, a diverse patient population is served by the JHHS, due to the large local catchment area as well as domestic and international referrals for care.

### 2.1. Study Population

EPIC SlicerDicer was used to identify adult patients aged 18 years and older who were seen within JHHS within a five-year period (27 September 2013 to 27 September 2018) and received one or more medications of interest. Medications of interest were selected based on associations with pruritus in previous literature and frequency of usage in clinical practice [1,2,3]. Sequential criteria were used to specify the subpopulation who presented with a chief complaint of “pruritus” or diagnosis (visit or billing) of “itching” within three months of receiving medication, using the systematized nomenclature of medicine—clinical terms (SNOMED-CT). Chronic diagnoses of pruritus or itching were excluded to rule out other causes of itch unrelated to new medication. Three months was chosen as the time period of interest to allow for the expected delay between development of drug-induced pruritus and access to care where symptoms could be clinically documented. Among patients with pruritus, the SNOMED-CT term, “eruption of the skin”, was used to identify those who developed an accompanying skin eruption in the same time period. “Eruption of the skin” is a parent term for most widespread skin diseases including rash, other nonspecific eruption of skin, and generalized drug eruption. The control population was comprised of adult patients aged 18 years and older seen in the same five-year period at JHHS who received a medication of interest, but without subsequent pruritus. Demographic variables including age, gender, and race were collected for both the study and control populations.

### 2.2. Medications of Interest

The three main drug classes queried were antimicrobial, cardiovascular, and psychiatric/neurologic drugs. For antimicrobials, numbers of patients developing subsequent pruritus with and without drug eruption were recorded for penicillin antibiotics, cephalosporins (first through fifth generation), macrolides, quinolones, tetracyclines, metronidazole, trimethoprim-sulfamethoxazole, and anti-malarial medications. For cardiovascular drugs, data was collected on angiotensin-converting enzyme (ACE) inhibitors, beta-blockers, calcium channel blockers, hydrochlorothiazide, amiodarone, heparin, and statins. For psychiatric/neurologic drugs, tricyclic antidepressants (TCAs), selective serotonin reuptake inhibitors (SSRIs), anti-epileptics, and opioid analgesics were investigated. Because of the smaller sample size of individual drugs, different drugs within the same category were grouped for increased power. For example, anti-epileptics were analyzed as an aggregate group and included carbamazepine, fosphenytoin, oxcarbazepine, phenytoin, and topiramate.

### 2.3. Statistical Analysis

Chi-squared tests were used to assess differences in proportions of categorical demographic variables (age, gender, and race) and rates of pruritus among different drug categories in Stata/IC15.1 (StataCorp, College Station, TX, USA). Pairwise student *t*-tests were also used to compare demographic variables, with a Bonferroni correction for multiple comparisons applied (*p* < 0.003 considered statistically significant). Frequencies of pruritus after receiving medication and of drug eruptions in those with pruritus were calculated using Microsoft Excel software (Microsoft Corporation, Redmond, WA, USA).

## 3. Results

### 3.1. Demographics

Of the patients studied, 9,802 developed pruritus, while 1,085,404 did not in the three-month period following initiation of the drug (Table 1). A higher proportion of patients with pruritus were female (70%) than those without (58%) (*p* < 0.001). Patients with pruritus were also more likely to be between 18–39 years of age compared to those without pruritus (*p* < 0.001). Patients in both groups were most commonly aged 50 to 79 years, but a greater percentage of patients with pruritus fell in this age group compared to those without, *p* < 0.001. A higher proportion of patients with pruritus were black (40%) as compared to patients without pruritus (23%), *p* < 0.001. In addition, a lower proportion of patients with pruritus were white or Native Hawaiian/Pacific Islander, respectively, compared to those without (*p* < 0.001, *p* = 0.002). Of the medications queried, the most commonly prescribed were opioid analgesics (n = 592,255), statins (n = 316,196), and cephalosporins (n = 252,342) (Table 2). The least commonly prescribed were amiodarone (n = 18,357), anti-epileptics (n = 38,147), and tricyclic anti-depressants (n = 38,147).

### 3.2. Rates of Pruritus within Three Months of Receiving Drug

**Figure 1:**
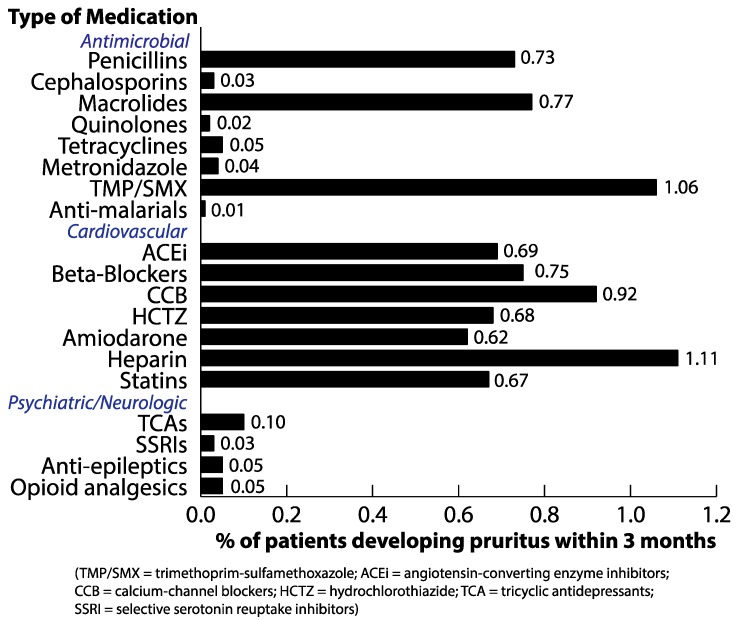
Frequency of pruritus after receiving drugs.

Rates of pruritus among drug classes queried were significantly different (*p* < 0.001). Among all drugs investigated, heparin (1.11%), trimethoprim–sulfamethoxazole (1.06%), and calcium channel blockers (0.92%) were associated with the highest rates of subsequent pruritus (Figure 1). In contrast, psychiatric/neurologic drugs as a class were associated with the lowest rates of subsequent pruritus: 0.1% in tricyclic anti-depressants, 0.03% in SSRIs, 0.05% in anti-epileptics, and 0.05% in opioid analgesics. Cardiovascular drugs were associated with generally higher rates of pruritus, with similar rates among ACE inhibitors (0.69%), beta-blockers (0.75%), hydrochlorothiazide (0.68%), amiodarone (0.62%), and statins (0.67%). Frequencies of subsequent pruritus were more varied among antimicrobial drugs. Higher rates of pruritus were associated with penicillin antibiotics (0.73%), macrolides (0.77%), and trimethoprim–sulfamethoxazole (1.06%) in contrast to relatively lower rates for cephalosporins (0.03%), quinolones (0.02%), tetracyclines (0.05%), metronidazole (0.04%), and anti-malarial drugs (0.01%).

### 3.3. Rates of Drug Eruption among Patients with Pruritus Subsequent to Receiving Drug

**Figure 2:**
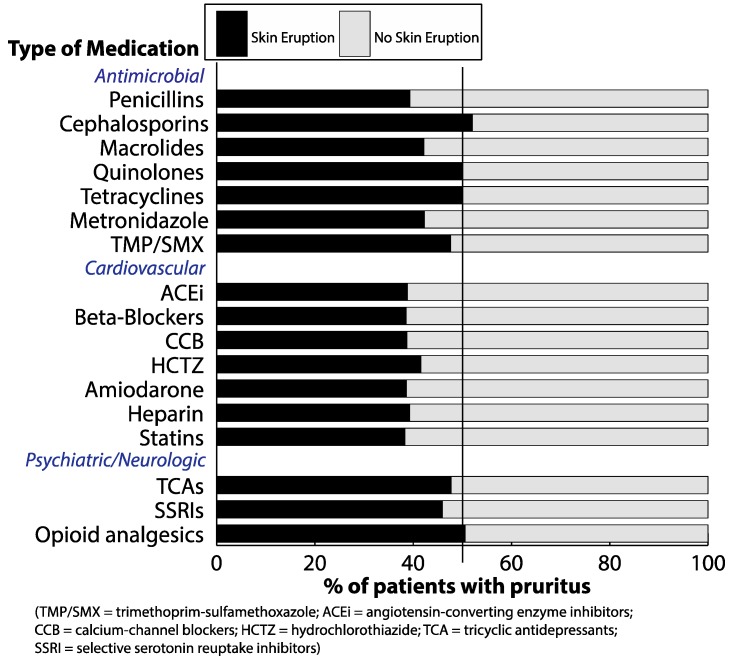
Frequency of skin eruption in patients with pruritus after receiving drugs.

In patients who developed pruritus, about half or fewer also developed skin eruption during the same time period (Figure 2). Rates of skin eruption in patients with pruritus were highest for cephalosporins (52.1%) and opioid analgesics (50.6%). Half (50.0%) of patients with pruritus who received quinolones and tetracyclines also developed skin eruptions, respectively. Rates of skin eruption among patients with pruritus after cardiovascular drugs were similar, ranging between 38.3% to 41.6% for all drugs queried. Frequencies of drug eruptions could not be reported for anti-malarial or anti-epileptic drugs, given the small absolute number of patients who experienced pruritus subsequent to initiation of these medications.

## 4. Discussion

Our findings confirm previous studies that have noted increased frequencies of drug-induced pruritus with certain antimicrobial and cardiovascular agents. Among antimicrobials, higher rates of pruritus with penicillin antibiotics and trimethoprim–sulfamethoxazole are thought to be secondary to inflammatory skin eruptions or cholestatic liver injury [1,3]. Among cardiovascular drugs, calcium channel blockers, beta blockers, and hydrochlorothiazide are associated with pruritus from skin inflammation, while itch with ACE inhibitors is thought to result from increased levels of bradykinin [3,6,7]. In addition, the rate of pruritus observed in statins (0.67%) is significant, considering its ubiquitous use as the second most commonly prescribed medication in the study (n = 316,196). Statin-induced xerosis cutis has been proposed as a potential mechanism of itch, with impaired barrier function resulting from decreased lipid distribution in the skin with inhibition of cholesterol biosynthesis [3]. In contrast, tricyclic antidepressants, SSRIs, and antiepileptics (including carbamazepine) were associated with low rates of pruritus. This is consistent with the established use of these agents to treat pruritus, through blockade of afferent neural pathways as well as direct action in the central nervous system [8].

Furthermore, we report that heparin was associated with a relatively high rate of pruritus (1.11%). This is in contrast to previous research noting rare heparin-induced itch primarily in the context of rare, IgE-mediated urticarial reactions [1]. The higher rate of heparin-induced pruritus in our study may capture a more frequent side effect of heparin: Pruritic, eczematous plaques at injection sites from delayed-type, non-IgE mediated allergic hypersensitivity (type IV) [9,10]. In contrast, we observed lower rates than previous studies of opioid-induced pruritus from triggering of non-immunological histamine release or effects on opioid receptors in the skin and central nervous system [11]. However, the frequency of opioid-induced pruritus is known to vary significantly—up to 50-fold—depending on the dosage, route of administration, and patient characteristics [12]. Lastly, macrolides have not been significantly implicated in previous literature of drug-related pruritus, but demonstrated a relatively high rate of subsequent pruritus in the present study (0.77%). Because of their significant anti-inflammatory properties, macrolides may have increased use in patients with chronic, inflammatory conditions that present episodically with pruritic flares [13]. It deserves further study whether macrolides also have the potential to invoke pruritus in individuals whose inflammatory condition may heighten their sensitivity to cutaneous side effects of medication [14].

To reduce confounding by indication, we compared relative rates of pruritus among patients who needed and received various drugs rather than to the healthier general population who did not. However, some residual confounding may remain as the drugs under study had a wide range of indications. Additional limitations of this study include its retrospective design and the use of aggregate data, which precluded multivariable analyses. Estimated rates of drug-induced pruritus may also be conservative and biased towards more severe reactions, given that cases were identified through clinical documentation of new itch with the Johns Hopkins Health System. Therefore, patients seen for drug-induced itch outside this network—such as at urgent care—may not be captured. Lastly, the small sample size of patients with pruritus limited our ability to perform specific subgroup analyses, despite evidence of ethnic differences in the development of itch (i.e., increased frequency with antimalarials in blacks) [15]. Furthermore, this also precluded analysis of demographics of patients with and without pruritus as stratified by inciting medication categories.

Of note, pre-existing pruritus in some patients improves upon initiation of antibiotics, but in this cohort, antibiotics were associated with subsequent pruritus [13]. Potential co-morbidities in these patients deserve further investigation, as tissue/nerve hypersensitivity in atopic or autoimmune conditions may contribute susceptibility to drug-induced pruritus [14]. We also observed a higher proportion of females in the group developing pruritus versus not (70% vs. 58%). This may be explained by pharmacokinetic and hormonal differences between genders that lead to differential drug metabolism, although their exact contribution to increased rates of adverse drug reactions in females is not well understood [16].

While overall rates of observed pruritus associated with medications were low (<1.2%), the relative frequency of pruritus with different medications within the same category (i.e., antimicrobials) varied up to 100-fold. Despite this significant magnitude of difference and the relatively higher rate of pruritus observed with certain medications, absolute correlation between these drugs and pruritus has not yet been proven. Future work in prospective studies should focus on elucidating drug-specific pathogenesis of itch to confirm these causal relationships. While difficult to quantify an exact threshold of pruritus frequency to establish high confidence of strong correlation between a certain drug and itch, the frequency threshold for clinical relevance is low. Given the high prevalence of these prescriptions in the patient population, even low absolute rates of drug-associated itch are clinically meaningful. Knowledge of relative rates of associated pruritus is therefore key to guiding provider selection of commonly used agents and minimizing adverse effects associated with reduced compliance.

## Figures and Tables

**Table 1:** Demographics.

Demographic	With Pruritus within 3 Months of Drug (n = 9802)	Without Pruritus After Receiving Drug (n = 1,085,404)	*p*-Value *	*p*-Value **
*Gender, (%)*				
Male	30.6	42.4	<0.001	<0.001
Female	69.4	57.6		<0.001
*Age, (%)*				
18–29	8.9	10.9	<0.001	<0.001
30–39	11.8	13.8		<0.001
40–49	12.6	13.5		0.009
50–59	19.1	17.6		<0.001
60–69	20.4	18.8		<0.001
70–79	16.3	14.8		<0.001
80–89	8.4	7.6		0.003
90–99	2.4	2.8		0.017
100+	0.1	0.2		0.027
*Race (%)*				
White/Caucasian	48.1	62.8	<0.001	<0.001
Black/African American	38.9	23.1		<0.001
Asian	4.2	4.3		0.627
American Indian/Alaska Native	0.4	0.3		0.072
Native Hawaiian/Pacific Islander	0.2	0.1		0.002
Other	7.7	7.4		0.259
Unknown	0.5	1.8		<0.001
Declined to answer	0.2	0.3		0.071

* Calculated by the chi-squared test for overall difference in proportions. ** Pairwise comparisons calculated using the student’s *t*-test with Bonferroni correction for significance (*p* < 0.003).

**Table 2:** Total number of patients who received drugs during study period.

Drug Type	Number Who Received Drug (N)
*Antimicrobial*	
Penicillins	177,487
Cephalosporins	252,342
Macrolides	124,856
Quinolones	128,248
Tetracyclines	87,497
Metronidazole	66,074
TMP/SMX	73,579
Anti-malarials	80,949
*Cardiovascular*	
ACEi	185,216
Beta-blockers	234,114
CCB	202,116
HCTZ	100,771
Amiodarone	18,357
Heparin	163,607
Statins	316,196
*Psychiatric/Neurologic*	
TCAs	43,756
SSRIs	212,244
Anti-epileptics	38,147
Opioid analgesics	592,255
